# Different Neurogenic Bladders in Patients with Cervical and Thoracic Myelopathy: Direct Comparison from a Prospective Case Series

**DOI:** 10.3390/jcm13144155

**Published:** 2024-07-16

**Authors:** Hyoungmin Kim, Bong-Soon Chang, Sanghyun Park, Yunjin Nam, Sam Yeol Chang

**Affiliations:** Department of Orthopedic Surgery, College of Medicine, Seoul National University Hospital, Seoul National University, Seoul 03080, Republic of Korea; hmkhm@snu.ac.kr (H.K.); bschang@snu.ac.kr (B.-S.C.); 7a794@snu.ac.kr (S.P.); namyunjin@snu.ac.kr (Y.N.)

**Keywords:** compressive myelopathy, cervical myelopathy, thoracic myelopathy, neurogenic bladder, urodynamic study

## Abstract

**Background/Objectives**: This study aimed to identify the unique characteristics of neurogenic bladders and associated symptoms in patients with cervical or thoracic myelopathy using clinical surveys and urodynamic studies (UDSs). **Methods**: Patients with degenerative cervical (DCM) or thoracic (DTM) myelopathy and lower urinary tract symptoms (LUTSs) scheduled for decompressive surgery were prospectively enrolled. A UDS was performed one day preceding surgery to evaluate the preoperative urological function. Subjective symptoms were evaluated using the International Prostate Symptom Score (IPSS) and the Japanese Orthopaedic Association Cervical Myelopathy Evaluation Questionnaire before surgery and one year postoperatively. **Results**: Sixty-two patients (DCM: 50, DTM: 12) with a mean age of 63.2 years (men: 30, women: 32) were enrolled. The UDS revealed that 5 (8.1%) patients, all with DCM, exhibited completely normal UDS results, and the remaining 57 had at least one abnormal finding. Based on the International Continence Society classification, an underactive bladder was significantly more common in patients with DTM compared to patients with DCM (75.0% vs. 18.0%, *p* < 0.001). The results of the questionnaire showed that the voiding symptom IPSS were significantly worse, preoperatively, in patients with DTM (5.0 ± 4.4 [DCM] vs. 8.7 ± 4.5 [DTM]; *p* = 0.013). One year postoperatively, the IPSS grade of 24.0% of patients with DCM improved, whereas only one (8.3%) patient with DTM showed improvement. **Conclusions**: Patients with DTM reported worse voiding symptoms and exhibited more underactive bladders on UDS than patients with DCM before decompression. One year postoperatively, more patients with DCM showed subjective improvements in urinary function than those with DTM.

## 1. Introduction

A neurogenic bladder (NB) can arise from various pathological conditions affecting the spinal column. Most prior research on NB focused on patients with spinal cord injury (SCI). Atraumatic compressive myelopathy can also cause urinary complaints due to NB, which is a common occurrence in the literature. Previous studies have reported that the prevalence of lower urinary tract symptoms (LUTSs) in surgically treated patients with degenerative cervical myelopathy (DCM) is up to 60% [1,2,3,4]. When a urodynamic study (UDS) is conducted in patients with DCM to diagnose NB, 20 to 50% of patients are diagnosed with NB [2,5]. Despite such a high prevalence of NB in patients with compressive myelopathy, there is limited evidence on how to diagnose and manage NB in these patients [6,7].

Two common types of NB are (1) an overactive bladder (OAB) during the storage phase and (2) an underactive bladder (UAB) during the voiding phase. An OAB due to detrusor overactivity causes storage symptoms such as frequency, nocturia, and urgency, whereas an UAB due to detrusor underactivity causes voiding symptoms such as a slow urinary stream, hesitancy, straining to void, and incomplete bladder emptying. Although these two types of NB have distinct clinical presentations and prognoses, few studies have described their patterns specifically in patients with compressive myelopathy [5].

Although some studies have reported the prevalence and clinical outcomes of NB in patients with DCM, studies on NB in patients with degenerative thoracic myelopathy (DTM) are rare [8,9]. Evidence is also limited regarding which type of NB is more common in patients with DTM and whether the distribution of OAB/UAB differs from that recorded in patients with DCM. Therefore, we conducted a prospective case series to determine the following: do patients with DCM and DTM exhibit different patterns of NB in terms of detrusor activity and recovery rates after decompressive surgery?

## 2. Materials and Methods

### 2.1. Study Design and Cohort

Consecutive patients with atraumatic cervical or thoracic compressive myelopathy and LUTS scheduled for decompressive surgery between April 2019 and November 2022 were prospectively screened for enrollment in this study. Only patients who agreed to undergo UDS before decompressive surgery were included. The Institutional Review Board of the author’s institution approved this prospective study (IRB No.: H-1902-136-1015), and all the participants provided written informed consent.

Patients with pre-existing urological disorders other than NB due to compressive myelopathy, such as benign prostatic hypertrophy (BPH) and stress urinary incontinence, were excluded after urological consultation. Patients with concomitant compressive myelopathy in the cervical and thoracic region, severe lumbar spinal stenosis (≥Schizas grade 3) [10,11], conus medullaris syndrome or cauda equina syndrome, and history of a previous spinal surgery who were unable to undergo UDS were also excluded. 

### 2.2. Urodynamic Study 

A UDS was performed one day preceding surgery to evaluate preoperative urological function. A UDS, consisting of free uroflowmetry with the measurement of post-void residual urine (PVR), urethral pressure profilometry, filling cystometry, and pressure-flow studies, was performed according to the guidelines of the International Continence Society (ICS). For the filling cystometry, normal saline at room temperature was infused at a rate of 50 mL/min, and detrusor activity, bladder sensation, capacity, and compliance during the storage phase were evaluated. Sphincter function was assessed using pelvic muscle electromyography. Bladder and sphincter functions during the voiding phase, including the presence of detrusor sphincter dyssynergia, were evaluated using fluoroscopy. A single independent urologist interpreted the UDS results and classified the neurogenic bladders based on the ICS classification (2017) [12]. 

### 2.3. Patient-Reported Outcomes

Preoperative urological symptoms were evaluated using the International Prostate Symptom Score (IPSS). The IPSS, initially developed by the American Urological Association to assess patients with BPH, is extensively validated and widely utilized in various urological disorders, including neurogenic bladders [13]. The IPSS classifies LUTS into three categories: (1) storage symptoms (questions 2, 4, and 7); (2) voiding symptoms (questions 3, 5, and 6); and (3) postmicturition symptoms (question 1). The IPSS provides scores for each LUTS category and the total IPSS, a simple summation of three categorical scores. In addition, three domains of the Japanese Orthopaedic Association Cervical Myelopathy Evaluation Questionnaire (JOACMEQ)—(1) bladder function, (2) lower-extremity function, and (3) quality of life (QOL)—were used to assess the functional status of the patients [14]. Postoperative symptom improvement was evaluated one year postoperatively using the same questionnaires.

### 2.4. Data Collection

We collected patient data after study enrollment and obtaining informed consent. Demographics, including age and sex, preoperative diagnosis, and symptom duration (from symptom onset to surgery), were collected before surgery. The severity of spinal cord compression was evaluated using the method described by Hirai et al. [15]. The spinal cord compression ratio was defined as the anterior–posterior (AP) diameter of the spinal cord at the most severely compressed intervertebral disc level divided by the AP diameter of the spinal cord in the C2 mid-vertebral body level in midsagittal T2-weighted images. After surgery, the surgical level, method, and the occurrence of postoperative complications were also recorded. 

### 2.5. Statistical Analysis 

A power analysis was conducted to determine the required sample size for the current study using G-Power 3.1 [16], focusing on the proportion of patients with a UAB on preoperative UDS, which we assumed to be greater in patients with TM than in those with CM. The prevalence of UAB in patients with atraumatic compressive myelopathy was adopted from previous studies [8,17], and a 3:1 allocation ratio of CM to TM was selected based on our clinical experience. An analysis with a two-sided alpha of 0.05, a statistical power of 0.80, and a dropout rate of 10% yielded a sample size of 70 patients. 

Comparative tests were performed to identify the distinctive patterns of urological dysfunction in patients with CM and TM. Continuous variables were reported as the mean ± standard deviation (SD) and compared using Student’s *t*-test and the Mann–Whitney test for the group comparison of clinical scores. The paired *t*-test and the Wilcoxon signed rank test were used for the improvement in the clinical scores. The proportion of patients with specific UDS findings was compared using a crosstab analysis (chi-square and Fisher’s exact tests). All statistical analyses were performed using IBM SPSS Statistics (version 28.0; IBM Corp., Armonk, NY, USA).

## 3. Results

**Figure 1:**
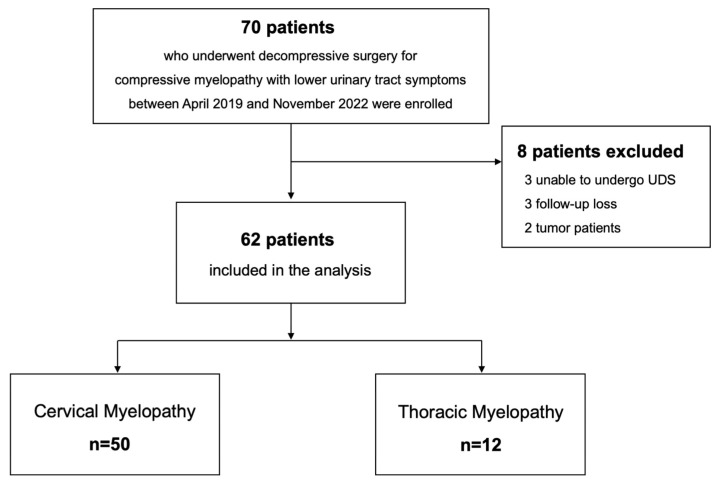
Study patients.

A total of 62 patients (30 men, 32 women) with a mean age of 63.2 years were included in the analysis (Figure 1). There were 50 patients with DCM and 12 with DTM in the cohort, and there were no significant differences in the demographics, symptom duration, and spinal cord compression ratio between the groups (Table 1). The preoperative diagnoses and surgical treatments of the patients with DCM and DTM are also summarized in Table 1. The average surgical levels were significantly longer in patients with DCM (1.7 ± 0.7 vs. 1.1 ± 0.3, *p* = 0.004). As for the surgical complications, there were three (6.0%) cases in the DCM group (one postoperative hematoma, one dysphagia, and one surgical-site infection), and one (0.8%) case of postoperative hematoma in the DTM group. 

Regarding UDS, five (8.1%) patients, all with DCM, exhibited completely normal UDS results despite having subjective LUTS. The remaining 57 had at least one abnormal UDS finding. Based on the ICS classification, the DCM group had more patients with OAB during the storage phase than the DTM group, although this difference was not statistically significant (Table 2). An underactive or acontractile bladder during the voiding phase was significantly more common in patients with TM than in those with DCM (75.0% vs. 18.0%, *p* < 0.001). Patients with a post-void residual urine volume > 50 mL were more common among those with DTM, although the difference was not statistically significant (33.3% vs. 20.2%).

A comparison of the clinical scores between the two groups is summarized in Table 3. Preoperatively, the voiding symptom score (5.0 ± 4.4 [DCM] vs. 8.7 ± 4.5 [DTM]; *p* = 0.013) was significantly worse in patients with DTM, whereas the two groups showed no significant difference in the other IPSS values. For the preoperative JOACMEQ scores, patients with DTM had lower scores in all three categories, but the differences did not reach statistical significance.

**Figure 2:**
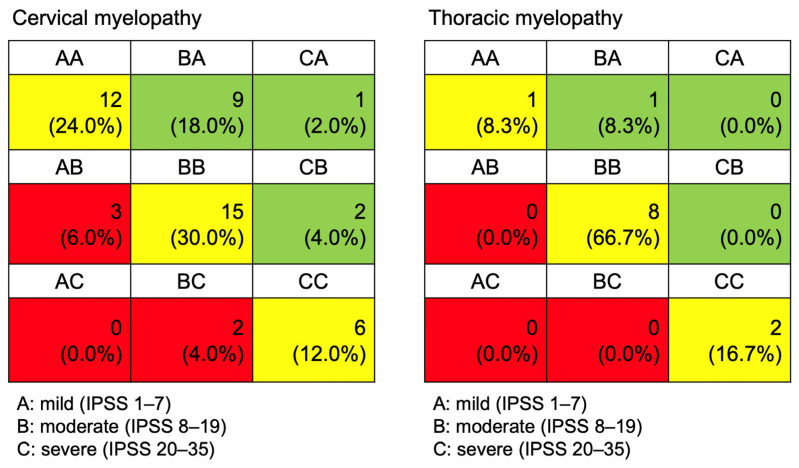
The changes in the IPSS grade from preoperative to one year postoperatively.

One year postoperatively, all three IPSS values, as well as the total IPSS, were worse in patients with DTM, although statistically insignificant (Table 3). Twelve (24.0%) patients in the DCM group showed improvement in the IPSS grade one year postoperatively, whereas only one (8.3%) patient with DTM showed improvement (Figure 2). Regarding the JOACMEQ scores, patients with DCM had a significantly higher QOL score than patients with DTM one year postoperatively (58.9 ± 22.9 vs. 43.2 ± 21.5, *p* = 0.031). The DCM group showed significant improvement in all three domains (bladder function, lower extremity, and QOL), whereas the DTM group did not show such improvements (Table 3).

When the total cohort was divided into overactive and underactive (including acontractile) bladders based on the preoperative UDS, regardless of the location of the compressive myelopathy, patients with an OAB had significant improvement in all domains of JOACMEQ one year postoperatively (Table 4). Meanwhile, patients with an underactive or acontractile bladder also showed some improvements in their JOACMEQ scores one year postoperatively, but the margin was smaller than that in patients with an OAB, and the improvement was not statistically significant. Except for one patient in the underactive bladder group who required CIC, all the patients in our study could self-void one year postoperatively.

## 4. Discussion

The current prospective study of patients with atraumatic compressive myelopathy undergoing decompressive surgery revealed different clinical features for NB in patients with DCM and DTM. More patients with DTM had underactive or acontractile bladders on their preoperative UDS and exhibited worse voiding IPSS values. One year postoperatively, the IPSS and JOACMEQ scores tended to be inferior in patients with DTM, and fewer patients in the DTM group showed an improvement in the IPSS grade of one or more.

In this study, 45 of the 50 (90.0%) patients with DCM and all 12 patients with DTM had at least one abnormal finding, especially NB, on preoperative UDS. Although this study only included patients with subjective LUTS, these rates are higher than those reported in previous studies. The rates of abnormal UDS in preoperative patients with DCM with subjective LUTS in previous studies ranged from 28.6% to 64.7% [2,5,17]. One of the reasons for the high rate of abnormal UDS in our cohort may be the difference in diagnostic criteria. We used the more recently introduced 2017 ICS classification [12], which is more specific than the previous version (2002) used in older studies [18]. Such a high rate of NB in preoperative UDS may also have resulted from the meticulous exclusion of patients with pre-existing urological disorders other than NB due to compressive myelopathy. In a study by Kim et al., the authors reported that six of their thirty-two patients had non-NB lower urinary tract dysfunction in the UDS [5]. There is a high possibility that these patients would have been excluded from our study. Therefore, we believe that our cohort, with such a high rate of abnormal UDS, is more appropriate for comparing the different features of NB between patients with DCM and DTM.

Regarding bladder function, 29.1% of the patients in our study showed an underactive or acontractile bladder on preoperative UDS. Theoretically, suprasacral spinal cord lesions, especially in patients with SCI, are associated with bladder overactivity because inhibition of the micturition reflex is lost [19]. However, bladder underactivity is also commonly observed in patients with atraumatic compressive myelopathy. Ando hypothesized that bladder underactivity can result from the compression of the posterior column in the spinal cord, affecting bladder proprioception, even in patients with DCM [20]. Sakakibara R et al. suggested that the pathway subserving detrusor activity is located in the lateral column of the spinal cord [21]. Regardless of a specific location in the spinal cord, based on clinical observations, it is evident that suprasacral spinal cord compression can cause detrusor underactivity without directly affecting the micturition centre in the conus medullaris.

This study aimed to compare the different features of NB between patients with DCM and DTM and found that patients with DTM had a higher proportion of underactive or acontractile bladders in their preoperative UDS (CM 18.0% vs. TM 75.0%). Similarly, patients with DTM had significantly worse voiding IPSS values preoperatively. These results are valuable because very few studies have described and compared the proportion of patients with bladder underactivity in patients with atraumatic DCM and DTM. In patients with SCI, Farrelly et al. reported that the proportion of UAB was significantly higher in those with thoracic SCI than cervical SCI (27/96, 28.1% vs. 2/63, 3.1%) [22]. Such a higher proportion of patients with bladder underactivity in the DTM group can be explained by the difference in the relative proportion of cross-sectional area that the pathway subserving detrusor activity occupies in the spinal cord. Because the proportion of pathways which subserve detrusor activity in the thoracic spinal cord is larger than that in the cervical spinal cord, patients with DTM may have more detrusor underactivity when the same degree of spinal cord compression is present. Our result that the two groups had a similar spinal cord compression ratio in the preoperative MRI further supports this theory (Table 1).

In our study, not only did the DTM group have worse IPSS values preoperatively, but they also had a lower proportion of patients with an improvement in their IPSS grade of one or more one year postoperatively (Figure 2). A study by Poublon et al., which investigated changes in the bladder-emptying method in 1403 patients with SCI who had been admitted for rehabilitation after SCI, reported similar results. In their study, the number of patients who were able to self-void normally at the time of hospital discharge was significantly greater in patients with cervical SCI compared to patients with thoracic SCI (63.8% vs. 48.0%, *p* < 0.01) [23]. This lack of urological improvements may be due to a higher percentage of underactive or acontractile bladder in DTM patients, as verified in our study.

Regarding the association between NB and motor function improvement in patients with compressive myelopathy, previous studies have reported that patients with urological dysfunction have a significantly limited recovery of lower-extremity motor function. In a study by Fukuda et al., the groups of patients with DCM with LUTS and positive NB on UDS had the lowest percentage of patients with significant lower-extremity function recovery as assessed by the JOACMEQ [2]. However, these studies did not specify bladder function and compared the recovery rate of lower-extremity function between patients with overactive and underactive bladders. Our results showed that patients with an underactive bladder had less improvement in their lower-extremity motor function (Table 4). Therefore, we can provide additional guidance to patients with compressive myelopathy on the prognosis of motor function recovery after surgery based on their preoperative UDS results.

The additional clinical significance that our study findings provide is that a preoperative UDS assessment can lead to early intervention by the urologist, which may improve clinical outcomes in urinary function. These interventions include medications, such as bethanechol chloride, which promotes bladder contractions, and timely CIC education to avoid unnecessary urinary retentions postoperatively. Except for one patient who required CIC, all the patients in our study could self-void one year postoperatively, which may be the result of early urological consultation and timely interventions.

This study had several limitations. First, although we conducted a power analysis to estimate a sufficient sample size to compare bladder activity on UDS between DCM and DTM, the sample size of this study may have been too small to derive statistical significance in the secondary outcomes. If we had a larger sample size, some outcomes that were not statistically significant could have differed. Second, we only examined the clinical outcomes one year postoperatively; long-term outcomes were not evaluated. Third, we cannot exclude the possibility of differences in the responsiveness to the IPSS between patients with overactive and underactive bladders, which may have led to the differences in recovery rate evaluated by the IPSS. The psychometric properties of the IPSS in patients with neurogenic bladders have not been investigated in previous studies and should be the topic of future studies. Fourth, we did not perform routine follow-up MRI examinations to evaluate the sufficiency of surgical decompression in this study, which is a potential factor associated with the recovery rate after surgery. Finally, urological function recovery one year postoperatively was evaluated only by questionnaires and not by UDS, as we considered it unethical to perform additional UDS for research purposes. Nevertheless, the current study provides novel and valuable descriptive information on urological dysfunction and recovery in patients with compressive cervical and thoracic myelopathies.

## 5. Conclusions

In this prospective study, patients with DTM complained of worse voiding symptoms and showed more UAB in the UDS than patients with DCM before decompressive surgery. One year postoperatively, more patients with DCM had significant improvements in their urinary function than patients with DTM.

## Figures and Tables

**Table 1:** Comparison of demographics, preoperative diagnosis, and surgical treatment.

	Cervical Myelopathy (*n* = 50)	Thoracic Myelopathy (*n* = 12)
Age (mean, SD)	62.9 ± 10.9	64.6 ± 9.9
Sex (M:F)	27:23	3:9
BMI (mean, SD)	26.3 ± 3.7	26.5 ± 2.6
Symptom duration (months)	14.8 ± 12.5	13.8 ± 8.8
Spinal cord compression ratio	0.58 ± 0.11	0.60 ± 0.09
Preoperative diagnosis	Cervical spondylotic myelopathy (*n* = 23) Cervical OPLL (*n* = 16) Cervical HIVD (*n* = 9) Atlantoaxial instability (*n* = 2)	Thoracic OYL (*n* = 6) Thoracic OPLL (*n* = 4) Thoracic HIVD (*n* = 2)
Surgical treatment	Cervical laminoplasty (*n* = 27) Anterior cervical discectomy and fusion (*n* = 17) Cervical laminectomy and fusion (*n* = 3) Atlantoaxial fusion (*n* = 2) Anterior cervical corpectomy and fusion (*n* = 1)	Posterior decompression and fusion (*n* = 10) Posterior decompression only (*n* = 2)

Abbreviations: BMI, body-mass index; HIVD, herniated intervertebral disc; OPLL, ossification of posterior longitudinal ligament; and OYL, ossification of yellow ligament.

**Table 2:** Comparison of preoperative urodynamic study findings *.

			Total (*n*, %)	Cervical Myelopathy (*n*, %)	Thoracic Myelopathy (*n*, %)	*p*-Value
Storage phase	Bladder function	Overactive	34 (54.8%)	30 (60.0%)	4 (33.3%)	0.354
	Bladder sensation	Hypersensitive	30 (48.4%)	24 (48.0%)	6 (50.0%)	0.539
		Hyposensitive	15 (24.2%)	11 (22.0%)	4 (33.3%)	0.258
	Bladder capacity	Above normal	19 (30.6%)	14 (28.0%)	5 (41.7%)	0.280
		Below normal	25 (40.3%)	22 (44.0%)	3 (25.0%)	0.524
	Urethral function	Incompetent	7 (11.3%)	5 (10.0%)	2 (16.7%)	0.612
Voiding phase	Bladder function	Underactive	14 (22.6%)	9 (18.0%)	5 (41.7%)	0.152
		Acontractile	4 (6.5%)	0 (0.0%)	4 (33.3%)	0.001
	Urethral function	Abnormal	13 (21.0%)	12 (24.0%)	1 (8.3%)	0.431

* The terminology and diagnostic criteria are based on the International Continence Society (ICS) classification (2017).

**Table 3:** Comparison of IPSS and JOA scores between patients with cervical and thoracic myelopathy.

		Preoperative			12 Months Postoperatively		
		Cervical	Thoracic	*p*-Value	Cervical	Thoracic	*p*-Value
IPSS	Storage symptom	5.2 ± 3.2	5.7 ± 4.0	0.912	4.3 ± 3.5	5.6 ± 3.9	0.288
	Voiding symptom	5.0 ± 4.4	8.7 ± 4.5	0.013	4.6 ± 4.7	6.5 ± 4.5	0.190
	Postmicturition symptom	1.8 ± 1.6	2.8 ± 1.9	0.108	1.8 ± 1.7	2.8 ± 1.9	0.120
	Total IPSS	12.0 ± 8.0	17.1 ± 8.7	0.057	10.8 ± 8.0	15.0 ± 7.3	0.086
JOA	Bladder function	68.4 ± 21.7	59.4 ± 19.1	0.133	76.6 ± 18.7 *	65.6 ± 16.3	0.051
	Lower-extremity function	52.7 ± 27.1	36.4 ± 25.4	0.060	66.3 ± 29.4 *	49.6 ± 30.0	0.099
	Quality of life	43.6 ± 15.5	35.6 ± 25.1	0.164	58.9 ± 22.9 *	43.2 ± 21.5	0.031

* Significant improvement from preoperative to 12 months postoperatively (*p* < 0.05).

**Table 4:** Comparison of clinical scores between overactive bladder and underactive bladder in preoperative UDS.

		Overactive Bladder (*n* = 34)			Underactive Bladder (*n* = 18)		
		Preop	12 Months Postop	*p*-Value *	Preop	12 Months Postop	*p*-Value *
IPSS	Storage	5.1 ± 3.3	4.1 ± 3.6	0.072	5.3 ± 3.4	6.3 ± 4.3	0.442
	Voiding	5.1 ± 4.4	5.1 ± 4.5	0.969	6.7 ± 4.6	5.8 ± 4.8	0.181
	Postmicturition	1.8 ± 1.6	1.6 ± 1.5	0.476	1.8 ± 1.8	2.8 ± 1.9	0.122
	Total score	11.9 ± 8.4	10.8 ± 7.5	0.303	13.8 ± 8.9	14.8 ± 8.5	0.669
JOA	Bladder function	67.7 ± 21.4	75.4 ± 20.0	0.034	69.4 ± 23.9	71.2 ± 19.4	0.307
	Lower extremity	46.7 ± 23.6	61.1 ± 28.1	0.001	53.8 ± 31.1	63.6 ± 35.1	0.148
	Quality of life	41.4 ± 17.4	55.0 ± 24.2	0.003	42.5 ± 23.8	55.3 ± 25.5	0.098

* *p*-values from the comparison between preoperative and 12-months postoperatively scores.

## Data Availability

All relevant raw data from the data presented in the manuscript are available from the authors of this study upon request.
